# Translational Up-Regulation and High-Level Protein Expression from Plasmid Vectors by mTOR Activation *via* Different Pathways in PC3 and 293T Cells

**DOI:** 10.1371/journal.pone.0014408

**Published:** 2010-12-28

**Authors:** Prashanthi Karyala, Nima D. Namsa, Durga Rao Chilakalapudi

**Affiliations:** Department of Microbiology and Cell Biology, Indian Institute of Science, Bangalore, India; New Mexico State University, United States of America

## Abstract

**Background:**

Though 293T cells are widely used for expression of proteins from transfected plasmid vectors, the molecular basis for the high-level expression is yet to be understood. We recently identified the prostate carcinoma cell line PC3 to be as efficient as 293T in protein expression. This study was undertaken to decipher the molecular basis of high-level expression in these two cell lines.

**Methodology/Principal Findings:**

In a survey of different cell lines for efficient expression of platelet-derived growth factor-B (PDGF-B), β-galactosidase (β-gal) and green fluorescent protein (GFP) from plasmid vectors, PC3 was found to express at 5–50-fold higher levels compared to the bone metastatic prostate carcinoma cell line PC3BM and many other cell lines. Further, the efficiency of transfection and level of expression of the reporters in PC3 were comparable to that in 293T. Comparative analyses revealed that the high level expression of the reporters in the two cell lines was due to increased translational efficiency. While phosphatidic acid (PA)-mediated activation of mTOR, as revealed by drastic reduction in reporter expression by n-butanol, primarily contributed to the high level expression in PC3, multiple pathways involving PA, PI3K/Akt and ERK1/2 appear to contribute to the abundant reporter expression in 293T. Thus the extent of translational up-regulation attained through the concerted activation of mTOR by multiple pathways in 293T could be achieved through its activation primarily by the PA pathway in PC3.

**Conclusions/Significance:**

Our studies reveal that the high-level expression of proteins from plasmid vectors is effected by translational up-regulation through mTOR activation via different signaling pathways in the two cell lines and that PC3 is as efficient as 293T for recombinant protein expression. Further, PC3 offers an advantage in that the level of expression of the protein can be regulated by simple addition of n-butanol to the culture medium.

## Introduction

Gene expression in mammalian cells can be regulated at a single or multiple levels involving chromatin structure, transcription, post-transcription and translation leading to different genes being expressed at widely varying levels in a cell type-specific manner or in the same cell. Functional expression of a gene can further be regulated by a multitude of post-translational mechanisms.

Currently, a very limited number of mammalian cell lines amenable for efficient transfection and expression of proteins is available. In contrast to lower eukaryotes or prokaryotes, mammalian cells provide biologically active proteins with relevant post-translational modifications. Unlike the tedious process involving transfection, selection, isolation and characterization of cell clones for expression by stable transfection of plasmid vectors, expression by transient transfection provides a rapid means for obtaining high concentrations of recombinant proteins. The human embryonic kidney-derived HEK293 cells [1] exhibit very high transfection efficiency and express the recombinant proteins at high levels [2], [3]. These cells were further modified by stable expression of the SV40 large T antigen generating the HEK293T (293T) cell line [4] which allows high level expression of proteins through episomal amplification of plasmids that contain SV40 origin of replication. The COS cells generated by immortalization of the African Green Monkey kidney cell line CV1 with replication-defective SV40 genome producing the large-T antigen have also been widely used for expression of recombinant proteins [5]. However, the versatility of these systems is limited by the use of vectors containing the relevant viral promoter and origin of replication. Chinese Hamster Ovary (CHO) cells are also widely used for stable expression of proteins, but are inefficient in protein expression by transient transfection [6]. The finding of the human cytomegalovirus major immediate early promoter as a powerful and versatile enhancer-promoter unit for expression vectors in a broad range of mammalian cells has obviated the need for specific viral promoter-replication origin-based vectors which have limited ability to drive expression in many cell lines [7]. Though 293T cells efficiently express genes from CMV promoter-driven vectors, there is a need to identify other cells that exhibit broader expression properties to express proteins that may not be expressed in 293T cells.

In a search for cell lines for high level expression of platelet-derived growth factor B (PDGF-B) from a transfected vector, the human prostate carcinoma cell line PC3 was found to be remarkably superior to many normal and tumour cell lines that were tested and the expression levels were on par with those in 293T. Since little is known on the mechanism/s underlying the high level expression from transfected vectors in 293T, it is of interest to carry out comparative analysis of the molecular mechanisms/signaling pathways that contribute to the high level expression phenotype in these two cell lines. Analysis of the mRNA and protein levels of the reporters in PC3, PC3BM, HeLa, MA104 and 293T revealed that the high-level expression of the reporters in PC3 is primarily due to enhanced translation. Since the mammalian target of rapamycin (mTOR) is the central controller of translation in mammalian cells, to understand the molecular basis for translational up-regulation in PC3, we first examined the status of activation of mTOR, its targets and other key translation regulators, and signaling pathways in PC3, and PC3BM and HeLa. Similar analysis between PC3 and 293T was also carried out to understand if the translational up-regulation in the two cell lines is effected through similar or dissimilar mechanisms. Our results reveal that while the high-level expression of the reporters in PC3 is mediated by activation of mTOR primarily through phosphatidic acid (PA) pathway, multiple mechanisms appear to potentiate the concerted up-regulation of translation in 293T.

## Results

### High level expression of recombinant proteins from transfected expression vectors in PC3 and 293T cells

PDGF-B gene expression *in vivo* is normally restricted to vascular endothelial cells [8], placental cytotrophoblasts [9], [10] and activated macrophages and monocytes [11], [12], but its expression is frequently deregulated in a variety of tumor cell lines [13]. During investigations on the role of the long 3′UTR of PDGF-B mRNA in cell type-specific expression or deregulation of its expression in tumor cells, PC3 was observed to express the PDGF-B protein from the transfected pCMV-PDGF-B vector at 5–50-fold higher level compared to all other cell lines that were used. It is to be noted that endogenous expression of PDGF-B mRNA or protein in PC3 is undetectable as in normal embryonic fibroblasts M413 and M426 (unpublished data).

**Figure 1:**
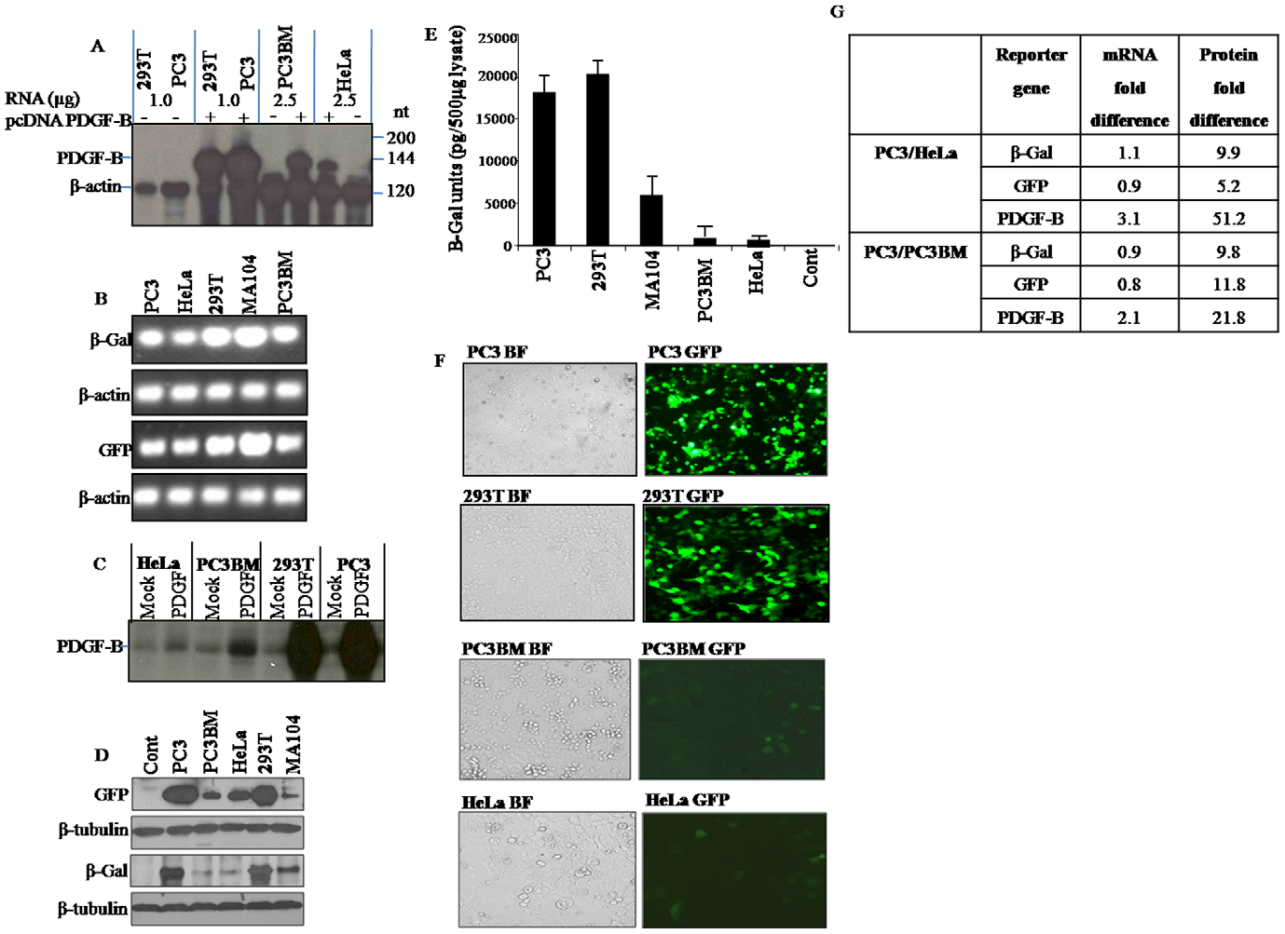
Analysis of RNA and protein levels derived from the transfected expression vectors of PDGF-B, GFP and β-Gal in PC3, PC3BM, HeLa, 293T and MA104 cells. (**A**) PDGF-B mRNA levels were determined by RNase protection Assay. The 144 nt protected band corresponds to PDGF-B and the 120 nt band represents that of β-Actin mRNA. (**B**) RT-PCR of β-Gal, GFP and β-Actin mRNA in pcDNA3-β-Gal and pcDNA3-GFP transfected cells. (**C**) Radioimmunoprecipitation of PDGF-B protein expressed in pCMV-PDGF-B transfected cells using an N-terminal antibody [69]. (**D**) Levels of β-Gal, GFP and β-Tubulin proteins in pcDNA3- β-Gal and -GFP transfected cells. 50 µg of transfected cell lysate was analyzed for GFP and β-Gal levels by SDS-PAGE. (**E**) β-galactosidase assay using the β-Gal ELISA Kit from Roche Diagnostics. (**F**) Fluorescence microscopy and bright field (BF) images of 293T, PC3BM, HeLa and PC3 cells transfected with pcDNA-GFP reporter gene construct. (**G**) Analysis of the fold differences in expression of the reporter mRNA and protein levels between PC3 and HeLa, and PC3 and PC3BM.

To investigate if the high-level expression of PDGF-B from transfected vector in PC3 is due to enhanced transcriptional, post-transcriptional or translational events, comparative analysis of RNA and protein levels of PDGF-B, β-gal and GFP from transfected vectors in PC3, PC3BM, HeLa, 293T and MA104 cells was carried out. As shown in figure 1A, the level of PDGF-B mRNA derived from pCMV-PDGF-B, as determined by RNase protection assay using 1.0 µg of RNA from PC3 and 293T and 2.5 µg from other cells, was higher in PC3 than in HeLa and PC3BM, but was slightly less than that in 293T. The levels of β-gal and GFP mRNAs in PC3, PC3BM and HeLa were similar but slightly higher in 293T and MA104 cells as estimated by semi-quantitative RT-PCR (Figure 1B). However, the level of PDGF-B protein as examined by radioimmunoprecipitation was very high in PC3 and 293T in comparison to HeLa and PC3BM (Figure 1C). Detection by western blotting of β-gal and GFP also revealed high level expression of the reporters in PC3 and 293T in comparison to other cell lines (Figure 1D), and their levels were similar in both PC3 and 293T (Figure 1C and 1D). These results were further confirmed by ELISA for β-gal (Figure 1E) and fluorescence microscopy for GFP (Figure 1F). Analysis of GFP fluorescence revealed that transfection efficiency in all the cells was similar. While the intensity of fluorescence is very high in PC3 and 293T, it was very weak in HeLa and PC3BM. The fold difference in GFP and β-gal mRNA levels in PC3 with respect to PC3BM and HeLa is approximately 1, though MA104 and 293T cells showed higher mRNA levels than PC3 (Figure 1B and 1G). But the levels of both reporter proteins were 5 to 10 fold higher in PC3 and 293T than in the other cell lines (Figure 1D and 1G). Thus the fold translational efficiency of the reporter mRNAs in PC3 and 293T was around 5 to 10 higher when compared to PC3BM and HeLa (Figure 1C, 1D, 1F and 1G). However, both RNA (2–3 fold) and protein (20–50 fold) levels of PDGF-B were higher in PC3 and 293T when compared to those in HeLa and PC3BM suggesting that the translational efficiency of PDGF-B mRNA in these cell lines is >10-fold than in other cells.

### Analysis of mTOR and its targets reveals hyperphosphorylation and inactivation of translational inhibitory factors in PC3

mTOR, a serine-threonine kinase, is the central controller of translation in mammalian cells [14]–[16]. mTOR exists in two structurally and functionally distinct multi-protein complexes called mTORC1 and mTORC2 [17]–[19]. Two upstream elements that regulate mTOR-signaling pathway include the Ras homolog enriched in brain (Rheb), a small GTP-binding protein, and the heterodimeric tuberous sclerosis complex 1 and 2 (TSC1/TSC2) [17]–[23]. TSC2, a GTPase activating protein (GAP) for Rheb, functions as a negative regulator of mTOR and facilitates the formation of the inactive GDP bound form of Rheb from the active GTP-bound form which activates mTOR kinase activity [22], [23]. Phosphorylation of TSC2-TSC1 by several signaling cascades leads to its inactivation, leading to mTORC1 activation [24]–[29]. Activated mTORC1 phosphorylates its two best-known downstream effectors, the 4E-binding proteins (4EBPs) and the ribosomal p70 S6 kinase (S6K1) [30]–[33]. Phosphorylation of these two translational regulators is frequently used as an *in vivo* readout for mTOR activation. 4EBP1 binds to the translation initiation factor eIF4E and prevents the formation of the eIF4F complex at the cap structure. Phosphorylated 4EBP1 fails to bind 4E, leading to recruitment of ribosomes to the mRNA and translational activation [34]–[36]. On the other hand, activated p70S6K1 phosphorylates the ribosomal protein S6 and eukaryotic elongation factor 2 kinase (eEF2K) leading to increased translational efficiency. Eukaryotic elongation factor 2 (eEF2) binds GTP and mediates the translocation step of translation elongation. Phosphorylation of eEF2 at T56 by eEF2K, within the GTP-binding domain, interferes with its ability to bind the ribosome [37]–[40]. Further, phosphorylation of eEF2K at S366 by activated S6K1 inhibits its kinase activity, leading to efficient translation [41], [42]. While the effects of mTORC1 are rapamycin sensitive, those of mTORC2 are rapamycin-insensitive which primarily regulates actin cytoskeletal polarization and reorganization [43],[44].

**Figure 2:**
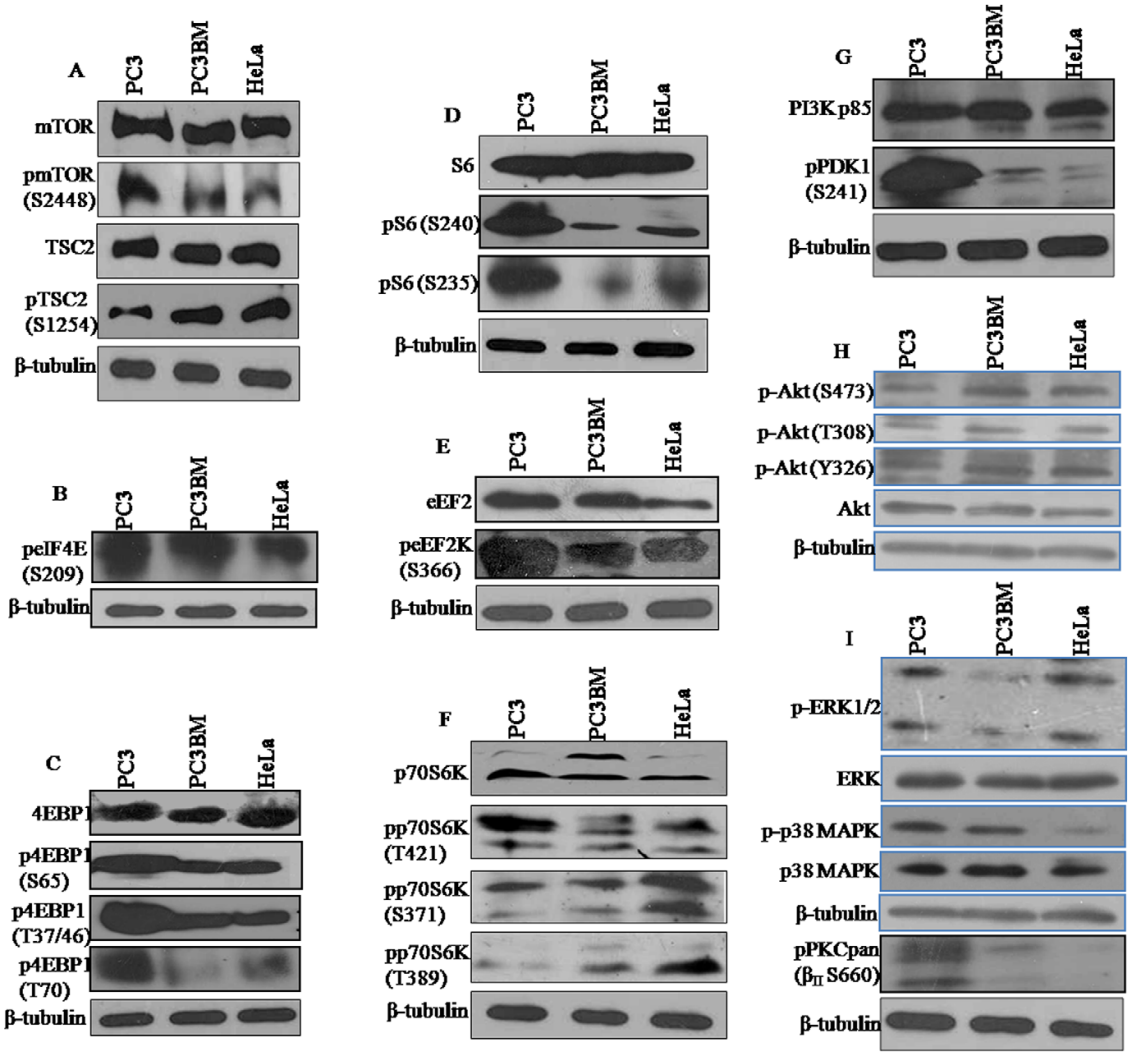
Western blot analysis of key target proteins of mTOR pathway. Analysis of total protein and/or phosphorylated forms of (**A**) mTOR and TSC2, (**B**) eIF4E, (**C**) 4EBP1, (**D**) S6, (**E**) eEF2 and eEF2K (**F**) p70S6K, (**G**) PI3K and PDK1, (**H**) Akt and (**I**) ERK1/2, p38 MAPK and PKC. 50 µg of cell lysate was used for analysis of mTOR, TSC2, PI3K, PDK1, Akt, eEF2, 4EBP1 and S6 and 100 µg was used for detection of phosphorylated forms and other proteins.

To understand the mechanism of 5–10-fold higher translational efficiency of the reporters in PC3 in comparison to PC3BM and HeLa, the status of mTOR, its regulators and the target translation factors in these three cell lines was examined. As shown in figure 2A, while the levels of mTOR and TSC2 were similar in the three cell lines, that of the phosphorylated TSC2 (S1254 phosphorylated by p38-activated kinase MK2 (MAPKAPK-2) [29] was less in PC3 than in the other cells. The activity of eIF4E, the limiting factor in translation initiation, is controlled in part by 4EBPs, [30]–[36], and by phosphorylation by Mnk1 [45], [46]. As shown in figure 2B, though the levels of phosphorylated forms of eIF4E were similar in PC3 and PC3BM but were relatively less in HeLa, 4EBP1 was hyperphosphorylated at all the four residues T37/46, T70 and S65 in PC3 than in the other cell lines, which is necessary to inhibit its interaction with eIF4E (Figure 2C). Further, the ribosomal protein S6 was hyper-phosphorylated at S240 and S235 in PC3 in comparison to PC3BM and HeLa. Though the level of the elongation factor eEF2 was similar in PC3 and PC3BM but higher than in HeLa (Figure 2E), the phosphorylated forms of the protein were not detectable in any of the cell lines using the available antibody (data not shown). However, eEF2K was significantly highly phosphorylated in PC3 than in the other two cell lines (Figure 2E).

### Analysis of the signaling pathways that regulate translation

PI3K-PKB/Akt, ERK MAPK pathways as well as p38-activated kinase MK2 function as the upstream positive regulators of mTORC1 through phosphorylation and inactivation of TSC2-TSC1 [24], [27], [29]. PI3K, activated by many growth signals, phosphorylates phosphatidylinositol-4, 5-bisphosphate (PIP2) at the 3-position to generate PIP3. PDK1 and PKB/Akt bind to PIP3 and are recruited to the membrane [47], [48] resulting in the phosphorylation of Akt by PDK1 and activation of Akt which leads to phosphorylation of both S6K and 4EBP1 [14], [15], [17], [19], [24]–[26], [36], [47], [48]. As shown in figure 2F, though p70S6K is hyperphosphorylated at T421 (by ERK MAPK pathways) in PC3, the level of phosphorylation at S371 and T389 (by mTOR) is significantly lower than that in HeLa, and HeLa and PC3BM, respectively (Figure 2F). While PDK1 is hyperphosphorylated at S241 in the activation loop in PC3, no significant differences in the levels of the p85 isoform of PI3K were observed (Figure 2G). Although no significant differences in the total Akt protein levels in the three cell lines were detected, the level of phosphorylation of the protein at Y326 (phosphorylated by Src), T308 (phosphorylated by PDK1) and S473 (phosphorylated by mTORC2 or PDK2) in PC3 was lower than that in PC3BM and HeLa (Figure 2H). Further, while PC3BM and HeLa significantly differed in the levels of the phosphorylated forms of ERK1/2 and p38 MAPK between themselves (Figure 2I) with the level of each kinase in either Hela or PC3BM being similar to that in PC3, the total protein levels of both the kinases are observed to be very similar in all the three cell lines. These observations suggest that PI3K-Akt and MAPK pathways are not major contributors of mTOR activation in PC3.

The lipid second messenger, phosphatidic acid (PA) is another important positive regulator of mTOR and protein synthesis [49], [50]. It mediates the mitogenic activation of mTOR signaling to the downstream effectors through regulation of a large number of protein kinases and phosphatases [49], [50]. PA is generated from phosphotidylcholine by phospholipase D (PLD) [50], [51]. Two mammalian isozymes of PLD, PLD1 and PLD2, have been identified to date [50], [51]. PLD1 has low basal activity, but can be activated by PIP_2_, PIP3 and several regulators including PKCs, small GTP binding proteins like RhoA, Rac1, ARF1, RalA, and Cdc42 and phosphatases [51]–[57]. PA-dependent translation activation occurs through the lipid messenger generated by PLD1 directly on mTOR (49–52, 55–58). mTOR can also be activated by diacylglycerol kinase-produced PA [58]. Though the mechanism of mitogen-dependent PLD2 activation is not fully understood, recent studies indicate PLD2-derived PA binds to and activates p70S6K1 in a rapamycin-insensitive and mTOR-independent manner [59]. PKCs regulate the activity of PLD either through direct interaction at the N-terminus or by phosphorylation [52]–[57]. While PKC and PLD regulate mTOR C1 signaling (49, 50, 60), mTORC2 is shown to control the folding and stability of Akt and conventional PKCs through phosphorylation of the turn motif (TM) and hydrophobic motif (HM) [19], [61], [62].

Analysis of the phosphorylation status of PKCs using the pPKC (pan) (β_II_ S660) antibody which recognizes PKC α, β_I_, β_II_, δ, ε, η and θ phosphorylated at the carboxy-terminal residue homologous to S660 of PKC β_II_ revealed that PKCs are considerably highly phosphorylated in PC3 than in PC3BM and HeLa (Figure 2I).

### Comparative analysis of the mTOR signaling Pathways in PC3 and 293T cells

**Figure 3:**
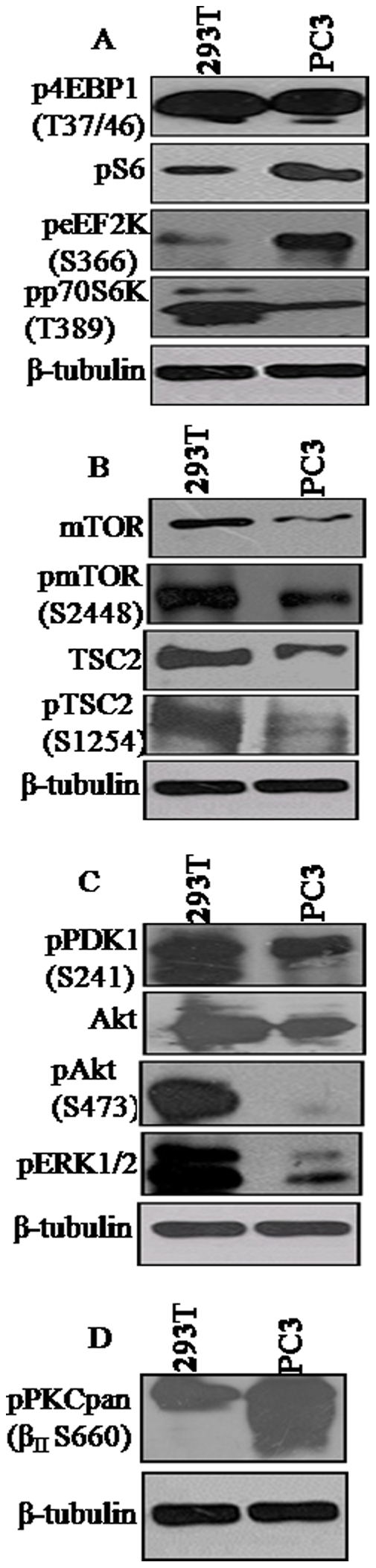
Comparative analysis of the translational regulatory proteins and different signaling pathway proteins in PC3 and 293 T cells. (**A**) Analysis of mTOR target proteins 4EBP1, S6, eEF2K and p70S6K. (**B**) mTOR and TSC2. (**C**) PI3K/Akt and ERK1/2. (**D**) PKC. 200 µg of cell lysate was used for PKC analysis and 50 µg was used for analysis of other proteins.

Since PC3 and 293T expressed the reporter RNA and protein at comparable levels (Figure 1), we sought to examine if the observed hyper-translation in these two cell lines is due to the activation of the same or different signaling pathways. Comparative analysis of the mTOR pathway target proteins, revealed that 4EBP1 is the only protein that showed comparable levels of hyperphosphorylation at T37/46 in both the cells lines (Figure 3A). While the levels of p70S6K-T389, mTOR, pmTOR-S2448, TSC2, pTSC2-S1254, pPDK1-S241, Akt, pAkt-S473 and pERK1/2 were considerably high in 293T than in PC3, by contrast, the phosphorylated forms of S6 and eEF2K (S366) were significantly higher in PC3 in comparison to 293T (Figure 3A, B and C). As revealed by the high levels of the phosphorylated forms of PDK1, Akt and ERK1/2 in 293T (Figure 3C), the two mTOR-activating signaling pathways, PI3K/Akt and Raf/MEK/ERK MAPK, appeared to be highly activated in 293T than in PC3. A remarkable observation was that PKCs were hyperphosphorylated in PC3 than in 293T (Figure 3D) suggesting that the two cell lines could significantly differ in the PA-mediated mechanism of mTOR activation.

**Figure 4:**
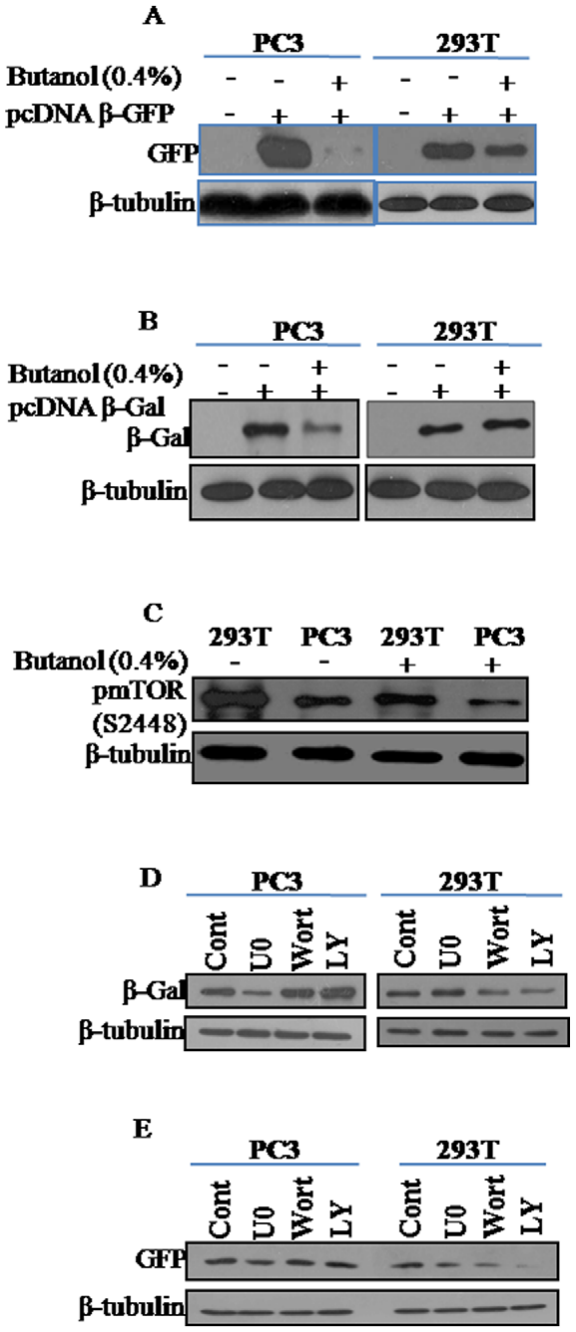
Analysis of the differential effect of n-Butanol, U0126, Wortmannin and LY294002 on reporter gene expression and phosphorylation of mTOR in transfected PC3 and 293T cells. Effect of n-butanol on expression of (**A**) GFP; (**B**) β-Gal, and (**C**) Inhibition of phosphorylation of mTOR by n-butanol; Effect of U0126 (U0), Wortmannin (Wort) and LY294002 (LY) on (**D**) β-Gal and (**E**) GFP expression. Proteins were separated by 12–14% SDS-PAGE and detected by immunoblotting using antibodies specific to GFP and β-Gal.

To assess the role of PA in the high-level expression phenotype of PC3, we examined the effect of n-butanol, an inhibitor of PA production, on reporter gene expression. PLD-mediated PA production *in vivo* can be blocked by primary alcohols like ethanol and n-butanol, which serve as a substrate in transphosphatidylation reaction by PLD to form phosphatidylethanol or phosphatidylbutanol [51]–[56]. Figure 4A and B illustrate that treatment of cells with 0.4% n-butanol had a profound inhibitory effect on both GFP and β-gal expression in PC3. However, though significant reduction in GFP expression was observed, butanol had no effect on β-gal expression in 293T cells (Figure 4B). n-butanol appears to have also affected the level of phosphorylation of mTOR in both cell lines (Figure 4C).

Since PI3K/Akt pathway appeared to have been highly activated in 293T than in PC3, their role in high level reporter expression in the two cell lines was evaluated using specific pharmacological inhibitors. While no inhibitory effect of PI3K/Akt inhibitors (Wotmannin, LY294002) on the expression of both the reporters was observed in PC3 (Figure 4D and 4E), translation of both reporters in 293T was inhibited by 50–75% with the expression of GFP being more severely affected compared to that of β-gal (Figure 4A and 4B). Though U0126 inhibited the expression of both the reporters significantly in PC3, its effect in 293T was again reporter specific. While there was no effect on β-gal expression in 293T, U0126 inhibited GFP expression by about 50% of that expressed in untreated cells. Thus the MEK/ERK pathway inhibitor U0126 appears to exhibit reporter-specific effects in 293T cells.

## Discussion

Comparative analysis of RNA and protein levels of the reporters in PC3, PC3BM, HeLa, MA104 and 293T cell lines indicated that the GFP and β-gal mRNAs are translated at 5–10 fold higher efficiency in PC3 and 293T (Figure 1C, 1D and 1G). It may be noted that though the CMV promoter appears to be relatively more active in MA104 cells, β-gal and GFP mRNAs are poorly translated than in PC3 and 293T. However, the PDGF-B mRNA levels were 2–3-fold higher in PC3 and 293T than in PC3BM and HeLa. Since the mRNA levels of GFP and β-gal transcribed from the CMV promoter in PC3, PC3BM and HeLa were similar, it is likely that the PDGF-B mRNA containing its long 3′UTR is selectively stabilized in PC3 and 293T. This aspect needs to be further investigated. Though the PDGF-B protein is expressed at 20–50-fold higher level in PC3 and 293T than in PC3BM and HeLa, the translational machinery in PC3 and 293T appears to be about 10–17-fold more efficient than in the other cells.

The fact that 4EBP1 is a direct target of mTOR and that 4EBP1 is hyperphosphorylated at all the 4 sites suggests that mTOR is more activated in PC3 in comparison to PC3BM and HeLa. Increased phosphorylation of mTOR at S2448 correlates with enhanced phosphorylation of S6K (T421) and 4EBP1 (Figure 2). Activation of p70 S6K1 depends on its level of phosphorylation state at eight sites: T229 (catalytic domain), S371, T389 and S404 (linker domain) and S411, S418, T421, and S424 (autoinhibitory domain) [63], [64]. T229 (phosphorylated by PDK1) plays a key role in modulating S6K1 activity. S6K1 activation may be achieved by sequential phosphorylation of these sites by MAPK pathways and PI3K [63], [64]. S6K1 activation up-regulates ribosome biosynthesis, and enhances cell translational capacity through phosphorylation of the 40S ribosomal protein S6 (rpS6) and upregulation of translation of mRNAs having 5′-terminal oligopyrimidine tracts (5′TOP), but in an mTOR-dependent, rapamycin–sensitive manner [36], [37], [65]. However, the low level phosphorylation of p70S6K at S371 and T389 in PC3 in comparison to PC3BM and HeLa does not correlate with the translation up-regulation in PC3 (Figure 2). It is possible that p70S6K could also be directly activated by PLD2-generated PA in PC3 [59].

Though hyperphosphorylation of PDK1 was observed, the lower levels of phosphorylation of Akt at 3 positions in PC3 than in PC3BM and HeLa, and the significant differences in the levels of the phosphorylated forms of both ERK and p38 MAPK kinases between PC3BM and HeLa suggest that these pathways do not significantly contribute to translational up-regulation in PC3. However, the MEK-ERK pathway appears to contribute, to some extent, to the high level expression of the reporters as seen by the reduced expression of GFP and β-gal by U0126 (Figure 4D and E).

The finding that PKCs (conventional and novel) are highly phosphorylated in PC3 than in PC3BM and HeLa (Figure 2I) and that mTOR phosphorylation at S2448 was higher with the Akt and phospho-Akt levels being either similar or lower than in PC3BM and HeLa suggest that mTOR in PC3 is primarily activated by PA-mediated mechanism. Further, the level of phosphoTSC2 (phosphorylated by PI3K-Akt and MAPK pathways) is significantly lower in PC3 than in the other two cell lines. The lack of inhibition of reporter expression by wortmannin and LY294002 (Figure 4) further suggests that PI3K-Akt pathway does not contribute to the translation up-regulation of reporters in PC3. Activation of PKCs requires phosphorylation in the activation loop (T500) by PDK1 [66] and our results indicate that PDK1 is hyperphosphorylated in PC3. Although PA can mimic mitogens in the activation of the mTOR downstream signaling pathway, PI3K is not activated in PA treated cells [49], but wortmannin blocks the action of PA suggesting that basal PI3K activity is required for PA to activate mTOR [38]. This suggests that basal PI3K activity, as seen by PDK1 phosphorylation, is required for activation of PKCs in PC3.

**Figure 5:**
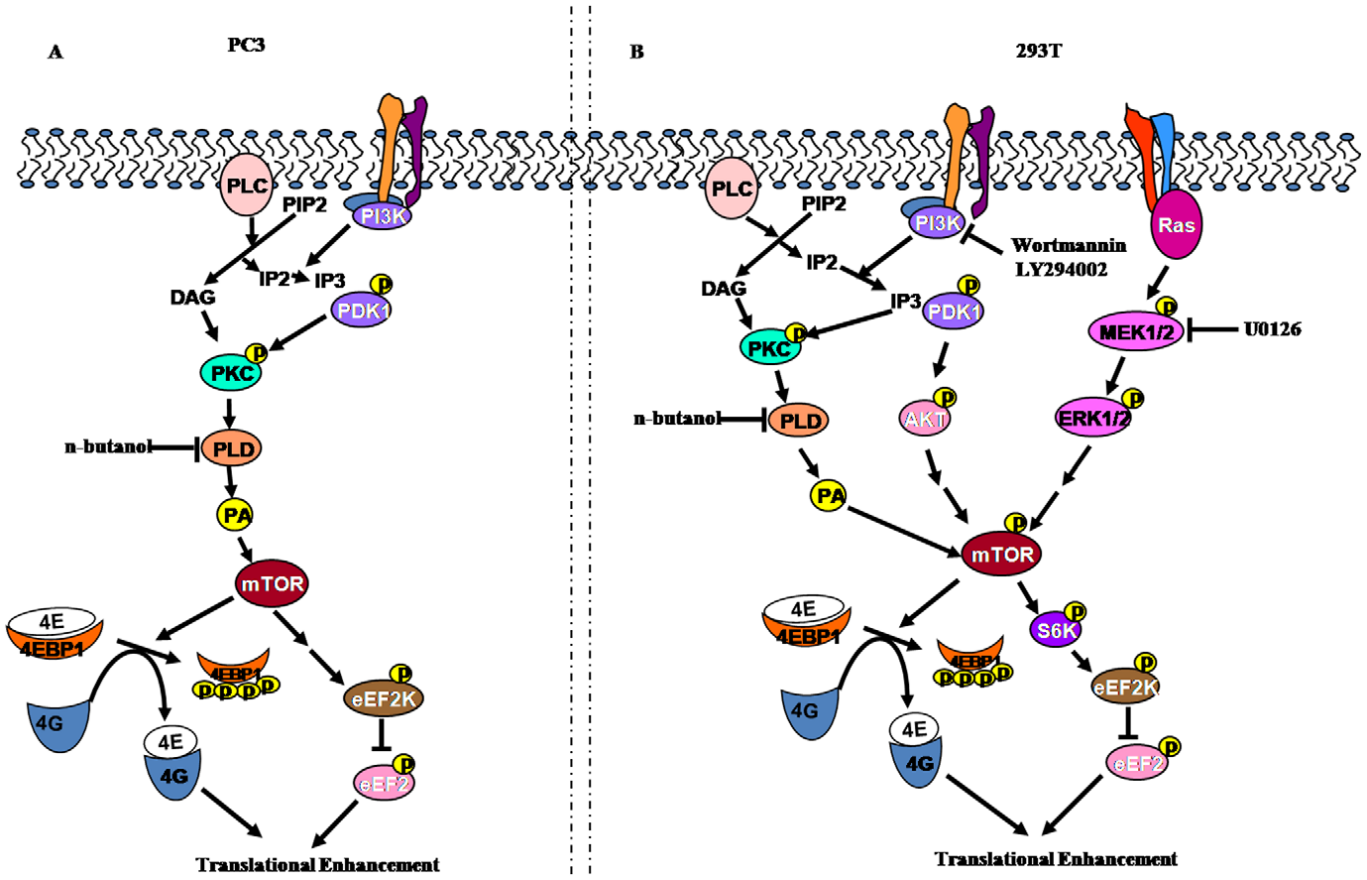
Schematic representation of activation of mTOR by common and distinct signaling pathways leading to translational up-regulation in (A) PC3 and (B) 293T cell lines.

Comparative analyses revealed that 293T and PC3 significantly differed in the activation of components of different signaling pathways. The only similarity that was observed between the two cell lines was in the level of 4EBP1 phosphorylation at T37/46. While the levels of phospho pKC (pan), S6 and eEF2K were significantly higher in PC3 than in 293T cells, the protein levels and/or the levels of the phosphorylated forms of p70S6K, mTOR, TSC2, PDK1 and Akt were notably higher in 293T cells than in PC3 (Figure 3). The higher levels of phospho TSC2 correlates with the robust activation of both the PI3K/Akt and ERK MAPK pathways in 293T than in PC3. The dramatic hyper-phosphorylation of PKCs in PC3 than in 293T indicates that efficient translation of reporters in PC3 is primarily mediated by PA-mediated mechanism while that in 293T is mediated by the concerted action of PI3K/Akt and MAPK pathways (Figure 5). The significant inhibition of reporter expression by wortmannin and LY294002 in 293T but not in PC3 in contrast to the severe inhibitory effect of n-butanol in PC3 in comparison to 293T further supports this conclusion. The PA-mediated pathway also appears to influence the high level expression in 293T as seen by the significant reduction in GFP but not β-gal expression by n-butanol (Figure 4A and 4B). U0126 also exhibited differential inhibitory effect on reporter expression in the two cell lines. While U0126 partially inhibited the expression of both reporters in PC3, it had no effect on β-gal expression in 293T. The reporter-specific effects observed with U0126 and n-butanol in 293T could be due to differences in the reporter mRNA and/or protein stability in the presence of the inhibitors. Our results suggest that inhibition of a single pathway could be partially compensated by other pathways and as such no drastic effect on reporter expression is observed by inhibition of any single pathway in 293T while PA-mediated pathway of translational up-regulation appears to be very crucial in PC3. In spite of the significant differences in the activation of different factors/pathways, both cells appear to have attained similar levels of enhanced translational capacity.

Our studies identify PC3 cell line to be as efficient as 293T in transfection efficiency and recombinant protein expression capacity. PC3 offers added advantage in that the level of protein expression from the transfected vector can be elegantly regulated by addition of different amounts of the inexpensive n-butanol to the culture medium.

## Materials and Methods

### Enzymes, reagents and oligonucleotides

AMV reverse transcriptase, restriction endonucleases, Taq DNA polymerase, T4 DNA ligase, protein molecular weight markers and other reagents were purchased from either Promega Biotech, Roche Applied Science, Invitrogen or Bio-Rad. The reporter-specific oligonucleotide primers were purchased from either Microsynth (Switzerland) or Sigma-Aldrich.

### Cell lines

The cell lines PC3, PC3BM, HeLa, 293T and MA104 were maintained in DMEM supplemented with 10% fetal bovine serum (FBS) (Invitrogen).

### Antibodies

Anti PDGF-B antibody was raised in the laboratory using MAP-peptide corresponding to the N-terminal region of PDGF-B [9], [67]. This antibody recognizes the PDGF-B polypeptide having an uncleaved N-terminus. Anti β-tubulin and GFP antibodies were from BD Biosciences and β-gal antibody was from Promega, respectively. All other antibodies were from Cell Signaling. The secondary anti-rabbit HRPO or anti-mouse HRPO antibodies were from GE Healthcare.

### Reporter gene construction

The pCMV-PDGF-B construct contains the PDGF-B cDNA region starting from nt 984–3373 that contains the open reading frame and the complete 3′ UTR followed by the 3′ genomic flanking sequence containing the 3′ processing signals [9] placed downstream of the cytomegalovirus (CMV) promoter. The Hind III-Dra I fragment of the β-gal gene from the vector pCH110 (Pharmacia) was subcloned in pBS (pBluescript, Stratagene) between the Hind III and EcoR V sites. The Hind III – BamH I β-gal fragment was then cloned downstream of the CMV promoter in pcDNA3.0 (Invitrogen) between the same sites to generate pcDNA-β-gal. The GFP open reading frame was PCR amplified from pF25-Rev-GFP [68] using primers mentioned below and subcloned in pBS between the EcoR V and Not I sites and was then inserted in pcDNA3.0 between the same sites generating pcDNA-GFP. The sequences of the primers used for GFP amplification are: 5′ primer 5′ATCGATATCCCGGGATGAGCAAAGCAGAAGAACTC-3′ and 3′ primer: 5′- AGTGCGGCCGCTCAGTTGTACAGTTCATCC -3′.

### Transfection and extraction of total RNA and protein

PC3, PC3BM, 293T, HeLa and MA104 cells grown to 50% confluency were transfected with three micrograms (µg) of vector DNA complexed with 6 µl of FuGENE 6 transfection reagent (Roche Applied Science) in 100 mm dish according to supplier's protocol. After 40–48 hours of transfection, total RNA or protein was extracted. Total RNA was extracted from the transfected cell lines using RNeasy minikit (Qiagen). For protein extraction, the cells were washed in tris-buffered saline (TBS) and lysed in 400 µl of ice-cold 1× RIPA buffer (50 mM Tris, 150 mM NaCl, 1% NP40, 0.5% sodium deoxycholate, 0.1% sodium dodecylsulphate (SDS) and 1 mM EDTA with protease inhibitor cocktail (Roche Applied Science). The lysates were centrifuged and protein concentration was determined by Bio-Rad Protein Assay reagent.

### Analysis of PDGF-B mRNA and protein expression

Total RNA, from the pCMV-PDGF-B-transfected PC3 (1.0 µg) and HeLa (2.5 µg), PC3BM (2.5 µg) and 293T (1.0 µg) cells, was analyzed by RNase protection assay using the RPA II kit from Ambion. The probe used for PDGF-B is from Bgl II site to the 4/5 exon junction spanning the position 1407–1548 nt [9] which yields a protected fragment of 141 nt in length. β-actin probe is prepared by transcription from pTRI-β-actin plasmid from Ambion using T7 RNA polymerase. This probe gives a protected fragment of size 121 nt.

For analysis of protein expression, 40 hours after transfection, cells were incubated for 30 min in medium lacking Met and Cys, followed by incubation for 4 hours in medium supplemented with 50 µCi each of [^35^S]-labelled Met and Cys. Cells were then lysed in 400 µl of RIPA buffer. About 800 µg of the labeled lysate was incubated with primary antibody (anti-N-terminal Ab against PDGF-B) [67], [69] for 1 hour, then 3–5 mg equivalent of Protein A Sepharose (swollen in RIPA) was added, incubated by continuous rotation at 4°C for 2 hours. The beads were washed thrice with RIPA buffer, Laemmli buffer was added, boiled for 10 min at 95°C and the proteins were resolved by 14% SDS-PAGE. The gel was fixed in 30∶15% methanol-acetic acid solution for 30 min, incubated in Enlightning Rapid Autoradiography Enhancer (Perkin Elmer) for 30 min, dried and exposed to X-ray film (Kodak XRP).

### RNA and Protein analysis of GFP and β-gal

RT-PCR was done using 0.3 µg of total RNA from β-gal and GFP-transfected cells using the Qiagen One step RT-PCR kit. β-gal and GFP mRNAs were amplified for 35 cycles and β-actin for 25 cycles. The forward and reverse primers used for GFP are: 5′-GCAACATACGGAAAACTTACCCTGA-3′and5′-CACTTTGATTCCATTCTTTTGTTTGTC-3′and those of β-gal are 5′-GAGCGAAAAATACATCGTCACCT-3′ and 5′-CAGGCAAAGCGCCATCGCCATTCA-3′. For protein analysis, PC3, PC3BM, HeLa, 293T and MA104 cells, grown in 100 mm cell culture dishes, were washed thrice in ice cold TBS and lysates were prepared in 600 µl of 1× RIPA buffer. Protein concentration was estimated using Bio-Rad Protein Assay reagent. Equal amounts of protein (50 µg) from different cells were analyzed by SDS-PAGE followed by western blot analysis using a chemiluminescence kit (Immobilon™ Western, Millipore).

### β-Galactosidase Assay

The cell extracts from the pcDNA-β-gal transfected cells were assayed for β-galactosidase activity using the β-gal ELISA kit from Roche Diagnostics.

### Fluorescence Microscopy

Cells transfected with pcDNA-GFP were examined using fluorescence Leica DM IRB microscope and the images were captured using Leica DC 300F digital camera, and were analyzed with the Leica IM500 Image Manager software.

### Analysis of translation factors in different cell lines

PC3, PC3BM, HeLa and 293T cells grown in 100 mm dishes were washed thrice in ice cold TBS and lysates prepared in cold 1× RIPA buffer (600 µl) containing 10 mM sodium pyrophosphate and 1 mM sodium orthovanadate and protease inhibitor cocktail. 50 µg of protein from different cell lines was used for analysis by SDS-PAGE and immunoblotting.

### Evaluation of the effect of n-butanol, Wortmannin, LY294002 and U0126 on the expression of β-gal and GFP reporters in PC3 and 293T cells

PC3 and 293T cells were grown to 50% confluency, media containing the serum was removed and the cells were pretreated or not treated with 0.4% n-butanol, Wortmanin (0.10 µM), LY294002 (30 µM) and U0126 (50 µM) (Cell Signaling) for 2 hours. The cells were then transfected for 4 hours with 3 µg of either pcDNA-GFP or pcDNA-β-gal DNA in fresh complete medium lacking the inhibitor. Cells were then grown in complete medium containing or lacking the compounds at indicated concentrations. β-gal and GFP expression was analyzed 40–48 hours after transfection.

## References

[1] Graham FL, Smiley J, Russell WC, Nairn R (1977). Characteristics of a human cell line transformed by DNA from human adenovirus type 5.. J Gen Virol.

[2] Schlaeger EJ, Legendre JY, Trzeciak A, Kitas EA, Christensen K, Merten OW, Perrin P, Griffiths B (1998). Transient transfection in mammalian cells.. New Developments and New Applications in Animal Cell Technology.

[3] Wurm F, Bernard A (1999). Large-scale transient expression in mammalian cells for recombinant protein production.. Curr Opin Biotechnol.

[4] Kim CH, Oh Y, Lee TH (1997). Codon optimization for high level expression of human erythropoietin (EPO) in mammalian cell- human or yeast codon usage effect on over-expression in 293T cell culture.. Gene.

[5] Gluzman Y (1981). SV40-transformed simian cells support the replication of early SV40 mutants.. Cell.

[6] Wurm FM (2004). Production of recombinant protein therapeutics in cultured mammalian cells.. Nat Biotechnol.

[7] Foecking MK, Hofstetter H (1986). Powerful and versatile enhancer-promoter unit for mammalian expression vectors.. Gene.

[8] Barrett TB, Gajdusek CM, Schwartz SM, McDougall JK, Benditt EP (1984). Expression of the sis gene by endothelial cells in culture and *in vivo*.. Proc Natl Acad Sci USA.

[9] Rao CD, Igarashi H, Chiu IM, Robbins KC, Aaronson SA (1980). Structure and sequence of the human c-sis/platelet-derived growth factor 2 (SIS/PDGF2) transcriptional unit.. Proc Natl Acad Sci USA.

[10] Goustin AS, Betsholtz C, Pfeifer-Ohlsson S, Persson H, Rydnert J (1985). Coexpression of the *sis* and *myc* proto-oncogenes in developing human placenta suggests autocrine control of trophoblast growth.. Cell.

[11] Barrett TB, Benditt EP (1987). sis (platelet-derived growth factor B chain) gene transcript levels are elevated in human atherosclerotic lesions compared to normal artery.. Proc Natl Acad Sci USA.

[12] Martinet Y, Bitterman PB, Mornex J-F, Grotendorst GR, Martin GR, Crystal RG (1986). Activated human monocytes express the c-sis proto-oncogene and release a mediator showing PDGF-like activity.. Nature.

[13] Igarashi H, Rao CD, Siroff M, Leal F, Robbins KC, Aaronson SA (1987). Detection of PDGF-2 homodimers in human tumor cells.. Oncogene.

[14] Hay N, Sonenberg N (2004). Upstream and downstream of mTOR.. Genes Dev.

[15] Gingras AC, Raught B, Sonenberg N (2001). Regulation of translation initiation by FRAP/mTOR.. Genes Dev.

[16] Schmelzle T, Hall MN (2000). TOR, a central controller of cell growth.. Cell.

[17] Bhaskar PT, Hay N (2007). The two TORs and Akt.. Dev Cell.

[18] Yang X, Yang C, Farberman A, Rideout TC, de Lange CFM, France J, Fan MZ (2008). The mammalian target of rapamycin signaling pathway in regulating metabolism and growth.. J Animal Sci.

[19] Inoki K, Guan K-L (2006). Complexity of the TOR signaling network.. Trends Cell Biol.

[20] Pearce LR, Huang X, Boudeau J, Pawlowski R, Wullschleger S (2007). Identification of Protor as a novel rictor-binding component of mTOR complex 2.. Biochem J.

[21] Garami A, Zwartkruis FJ, Nobukuni T, Joaquin MM, Roccio M (2003). Insulin activation of Rehb, a mediator of mTOR/S6K/4E-BP signaling, is inhibited by TSC1 and 2.. Mol Cell.

[22] Inoki K, Li Y, Xu T, Guan KL (2003). Rheb GTPase is a direct of TSC2 GAP activity and regulates mTOR signaling.. Genes Dev.

[23] Long X, Lin Y, Oritz-Vega S, Yonezawa K, Avruch J (2005). Rheb binds and regulates the mTOR kinase.. Curr Biol.

[24] Gingras AC, Kennedy SG, O'Leary MA, Sonenberg N, Hay N (1998). 4E-BP1, a repressor of mRNA translation, is phosphorylated and inactivated by the Akt (PKB) signaling pathway.. Genes Dev.

[25] Inoki K, Li Y, Zhu T, Wu J, Guan KL (2002). TSC2 is phosphorylated and inhibited by Akt and suppresses mTOR signaling.. Nat Cell Biol.

[26] Manning BD, Tee AR, Logsdon MN, Blenis J, Cantley LC (2002). Identification of the tuberous sclerosis complex-2 tumor suppressor gene product tuberin as a target of the phosphoinositide 3-kinase/Akt pathway.. Mol Cell.

[27] Ma L, Chen Z, Erdjument-Bromage H, Tempst P, Pandolfi PP (2005). Phosphorylation and functional inactivation of TSC2 by Erk: implications for tuberous sclerosis and cancer pathogenesis.. Cell.

[28] Roux PP, Ballif BA, Anjum R, Gygi SP, Blenis J (2004). Tumor-promoting phorbol esters and activated ras inactivate the tuberous sclerosis tumor suppressor complex via p90 ribosomal S6 kinase.. Proc Natl Acad Sci USA.

[29] Li Y, Inoki K, Vacratsis P, Guan KL (2003). The p38 and MK2 kinase cascade phosphorylates tuberin, the tuberous sclerosis 2 gene product, and enhances its interaction with 14-3-3.. J Biol Chem.

[30] Brunn GJ, Hudson CC, Sekulic A, Williams JM, Hosoi H (1997). Phosphorylation of the translational repressor PHAS-1 by the mammalian target of rapamycin.. Science.

[31] Burnett PE, Barrow RK, Cohen NA, Snyder SH, Sabatini DM (1998). RAFT1 phosphorylation of the translational regulators p70 S6 kinase and 4E-BP1.. Proc Natl Acad Sci USA.

[32] Schlam SS, Fingar DC, Sabatini DM, Blenis J (2003). TOS motif-mediated raptor binding regulates 4E-BP1 multisite phosphorylation and function.. Curr Biol.

[33] Nojima H, Tokunaga C, Eguchi S, Oshino N, Hidayat S (2003). The mammalian target of rapamycin (mTOR) partner, raptor, binds the mTOR substrates p70 S6 kinase and 4E-BP1 through their TOR signaling (TOS) motif.. J Biol Chem.

[34] Gingras AC, Raught B, Gygi SP, Niedzwiecka A, Miron M (2001). Heirarchical phosphorylation of the translation inhibitor 4E-BP1.. Genes Dev.

[35] Mothe-satney I, Yang D, Fadden P, Haystead TA, Lawrence JrJC (2000). Multiple mechanisms control phosphorylation of PHAS-1 in five (S/T)P sites that govern translation repression.. Mol Cell Biol.

[36] Proud CG (2004). Signaling to translation: How signal transduction pathways control the protein synthetic machinery.. Biochem J.

[37] Avruch J, Belham C, Weng Q, Hara K, Yonezawa K (2001). The p70 S6 kinase integrates nutrient and growth signals to control translational capacity.. Prog Mol Subcell Biol.

[38] Browne GJ, Proud CG (2002). Regulation of peptide chain elongation in mammalian cells.. Eur J Biochem.

[39] Browne GJ, Proud CG (2004). A novel mTOR-regulated phosphorylation site in elongation factor 2 kinase modulates the activity of the kinase and its binding to calmodulin.. Mol Cell Biol.

[40] Sans MD, Xie Q, Williams JA (2004). Regulation of translation elongation and phosphorylation of eEF2 in rat pancreatic acini.. Biochem Biophys Res Commun.

[41] Ryazanov AG, Ward MD, Mendola CE, Pavur KS, Dorovkov MV (1997). Identification of a new class of protein kinases represented by eukaryotic elongation factor-2 kinase.. Proc Natl Acad Sci USA.

[42] Wang X, Li W, Williams M, Terada N, Alessi D, Proud CG (2001). Regulation of elongation factor 2 kinase by p90 (RSK1) and p70 S6 kinase.. EMBO J.

[43] Wullschleger S, Loewith R, Hall MN (2006). TOR signaling in growth and metabolism.. Cell.

[44] Sarbassov DD, Ali SM, Kim DH, Guertin DA, Latek RR (2004). Rictor, a novel binding partner of mTOR, defines a rapamycin-insensitive and raptor-independent pathway that regulates the cytoskeleton.. Curr Biol.

[45] Pyronnet S, Imataka H, Gingras AC, Fukunaga R, Hunter T, Sonenberg N (1999). Human eukaryotic translation initiation factor 4G (eIF4G) recruits MNK1 to phosphorylate eIF4E.. EMBO J.

[46] Ueda T, Watanabe-Fukunaga R, Fukuyama H, Ngata S, Fukunaga R (2004). Mnk2 and Mnk1 are essential for constitutive and inducible phosphorylation of eukaryotic initiation factor 4E but not for cell growth or development.. Mol Cell Biol.

[47] Cantrell DA (2001). Phosphoinositide 3-kinase signaling pathways.. J Cell Sci.

[48] Newton AC (2009). Lipid activation of protein kinases.. J Lipid Res.

[49] Fang Y, Vilella-Bach M, Bachmann R, Flanigan A, Chen J (2001). Phosphatidic acid-mediated mitogenic activation of mTOR signaling.. Science.

[50] Jenkins GM, Frohman MA (2005). Phospholipase D: a lipid centric review.. CMLS Cell Mol Life Sci.

[51] Exton JH (2002a). Phospholipase D-structure, regulation and function.. Rev Physiol Biochem Pharmacol.

[52] Exton JH (2002b). Regulation of phospholipase D.. FEBS Lett.

[53] Cazzolli R, Shemon AN, Fang MQ, Hughes WE (2006). Phospholipid signaling through phospholipase D and phosphatidic acid.. IUBMB Life.

[54] Cockcroft S (2001). Signalling roles of mammalian phospholipase D1 and D2.. CMLS Cell Mol Life Sci.

[55] Houle MG, Bourgoin S (1999). Regulation of phospholipase D by phosphorylation-dependent mechanisms.. Biochim Biophys Acta.

[56] Hui L, Rodrik V, Pielak RM, Knirr S, Zheng Y, Foster DA (2005). mTOR-dependent suppression of protein phosphatase 2A is critical for phospholipase D survival signals in human breast cancer cells.. J Biol Chem.

[57] Brognard J, Newton AC (2008). PHLiPPing the switch on Akt and protein kinase C signaling.. Trends Endocrinol Metabol.

[58] Avila-Flores A, Santos T, Rincon E, Merida I (2005). Modulation of the mammalian target of rapamycin pathway by diacylglycerol kinase-produced phosphatidic acid.. J Biol Chem.

[59] Lehman N, Ledford B, Di Fulvio M, Frondorf K, McPhail LC, Gomez-Cambronero J (2007). Phospholipase D2-derived phosphatidic acid binds to and activates ribosomal p70 S6 kinase independently of mTOR.. FASEB J.

[60] Kumar V, Pandey P, Sabatini D, Kumar M, Majumder PK (2000). Functional interaction between RAFT1/FRAP/mTOR and protein kinase Cδ in the regulation of cap-dependent initiation of translation.. EMBO J.

[61] Facchinetti V, Ouyang W, Wei H, Soto N, Lazorchak A (2008). The mammalian target of rapamycin complex 2 controls folding and stability of Akt and protein kinase C.. EMBO J.

[62] Ikenoue T, Inoki K, Yang Q, Zhou X, Guan KL (2008). Essential function of TORC2 in PKC and Akt turn motif phosphorylation, maturation and signaling.. EMBO J.

[63] Dufner A, Thomas G (1999). Ribosomal S6 kinase signaling and the control of translation.. Exp Cell Res.

[64] Berven L A, Crouch MF (2000). Cellular function of p70^S6K^: A role in regulating cell motility.. Immunol Cell Biol.

[65] Patursky-Polischuk I, Stolovich-Rain M, Hausner-Hanochi M, Kasir J, Cybulski N (2009). The TSC-mTOR pathway mediates translational activation of TOP mRNAs by insulin largely in a raptor- or rictor-independent manner.. Mol Cell Biol.

[66] Le Good JA, Ziegler WH, Parekh DB, Alessi DR, Cohen P, Parker PJ (1998). Protein kinase C isotypes controlled by phosphoinositide 3-kinase through the protein kinase PDK1.. Science.

[67] Robbins KC, Antoniades HN, Devare SG, Hunkapillar MW, Aaronson SA (1983). Structural and immunological similarities between simian sarcoma virus gene product(s) and human platelet-derived growth factor.. Nature.

[68] Stauber RH, Horie K, Carney P, Hudson EA, Tarasova NI (1998). Development and applications of enhanced green fluorescent protein mutants.. Biotechniques.

[69] Rao CD, Pech M, Robbins KC, Aaronson SA (1988). The 5′ untranslated sequence of the *sis*/platelet-derived growth factor 2 transcript is a potent translational inhibitor.. Mol Cell Biol.

